# Quantum-Mechanical Assessment of the Energetics of Silver Decahedron Nanoparticles

**DOI:** 10.3390/nano10040767

**Published:** 2020-04-16

**Authors:** Svatava Polsterová, Martin Friák, Monika Všianská, Mojmír Šob

**Affiliations:** 1 Department of Chemistry, Faculty of Science, Masaryk University, Kotlářská 2, 611 37 Brno, Czech Republic; polsterova@mail.muni.cz (S.P.); 230038@mail.muni.cz (M.V.); mojmir@ipm.cz (M.Š.); 2 Central European Institute of Technology, CEITEC IPM, Institute of Physics of Materials, Czech Academy of Sciences, Žižkova 22, 616 62 Brno, Czech Republic; 3 Institute of Physics of Materials, Czech Academy of Sciences, Žižkova 22, 616 62 Brno, Czech Republic; 4 Central European Institute of Technology, CEITEC MU, Masaryk University, Kamenice 753/5, 625 00 Brno, Czech Republic

**Keywords:** nanoparticles, thermodynamics, silver, decahedron, excess energy, *ab initio* calculations

## Abstract

We present a quantum-mechanical study of silver decahedral nanoclusters and nanoparticles containing from 1 to 181 atoms in their static atomic configurations corresponding to the minimum of the *ab initio* computed total energies. Our thermodynamic analysis compares T = 0 K excess energies (without any excitations) obtained from a phenomenological approach, which mostly uses bulk-related properties, with excess energies from *ab initio* calculations of actual nanoclusters/nanoparticles. The phenomenological thermodynamic modeling employs (i) the bulk reference energy, (ii) surface energies obtained for infinite planar (bulk-related) surfaces and (iii) the bulk atomic volume. We show that it can predict the excess energy (per atom) of nanoclusters/nanoparticles containing as few as 7 atoms with the error lower than 3%. The only information related to the nanoclusters/nanoparticles of interest, which enters the phenomenological modeling, is the number of atoms in the nanocluster/nanoparticle, the shape and the crystallographic orientation(s) of facets. The agreement between both approaches is conditioned by computing the bulk-related properties with the same computational parameters as in the case of the nanoclusters/nanoparticles but, importantly, the phenomenological approach is much less computationally demanding. Our work thus indicates that it is possible to substantially reduce computational demands when computing excess energies of nanoclusters and nanoparticles by *ab initio* methods.

## 1. Introduction

The silver nanoparticles are widely used as antiviral agents [1,2], sensors [3,4], catalysts [5], as nanoparticle solders [6,7] as well as in numerous others application. Nanoclusters, as extreme cases of nanoparticles, have a yet greater surface/volume ratio and different geometries and electronic structures when compared with their bulk counterparts. Theoretical computations constitute a very advantageous tool when studying nanoclusters as they can accurately determine many of their characteristics, such as their surface type, strain energies [8,9], phase diagrams [10] or information on their catalytic activity. Many studies reported that modifications of the surface energy can change the shape of a (nano-)particle and/or its melting temperature [11,12,13]. The surface energy of a nanoparticle is often considered as the most important factor in catalysis, crystal growth, sintering and other surface-related processes. The most stable surface geometry for nanoparticles of pure fcc transition metals is the {111} facet [14] but the situation can differ in multi-component cases [15].

Relative to the bulk, the {111} facet exhibits the highest density of atoms and the highest coordination number of surface atoms. The most stable structures of fcc nanoclusters include the icosahedron, cuboctahedron and decahedron [8]. Another energy contribution is that related to strain. The strain energy of the particle can be affected by many factors. As the ratio of surface to volume decreases, the effect of surface stress is more significant and leads to the compression of particles [8].

**Figure 1:**
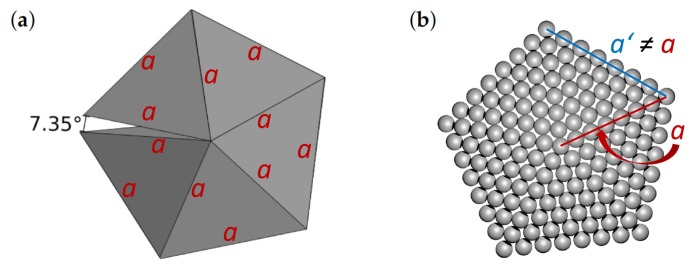
Schematic visualizations of (**a**) five tetrahedrons forming an imperfect decahedron with a gap of 7.35${}^{{}^{\circ}}$ and (**b**) one of the studied decahedral nanoclusters/nanoparticles (see below) without the gap. A characteristic length *a* defined here is used below when defining the shape factor.

Our study is focused on decahedral particles which have very interesting plasmonic and optical properties [16] as well as catalytic possibilities due to high strain energy [9]. The decahedron and icosahedron are inherently strained due to twinning and unfilled volume [17]. In particular, the decahedral nanoclusters are balancing the surface stability of five tetrahedrons (see Figure 1), which exhibit the {111} facets, against the strain energy related to an internal unfilled gap of 7.35° and distortion induced by their twinned internal structure [9]. The actual shape of the studied nanoparticles can deviate from a prediction by the Wulff construction due to the influence of the internal strain and strain-associated strain energy (in particular in the case of intermediate states [18,19]).

## 2. Methods

The energies of studied decahedral nanoclusters and nanoparticles were calculated in two different ways which are both connected with quantum-mechanical Density Functional Theory (DFT) [20,21] calculations. The first method is a phenomenological thermodynamic modeling based on the CALPHAD method when the energy of nanoclusters and nanoparticles is approximated by a sum of relevant energy contributions corresponding (i) to a defect-free bulk material and (ii) surface energies and stresses (related to surfaces of a bulk, not nanoparticles) [22,23,24,25,26]. It is customary now that some or all energy contributions used in CALPHAD approach are computed using quantum-mechanical methods. Let us note that the idea of connecting the CALPHAD method and *ab initio* calculations is not trivial. It was presented first before the end and at the beginning of the new millennium in papers [27,28,29,30,31,32] and has been used many times since then (see e.g., [33,34,35,36,37,38,39,40,41,42]), also in studies of nanoalloys and nanoparticles, as mentioned above.

**Figure 2:**
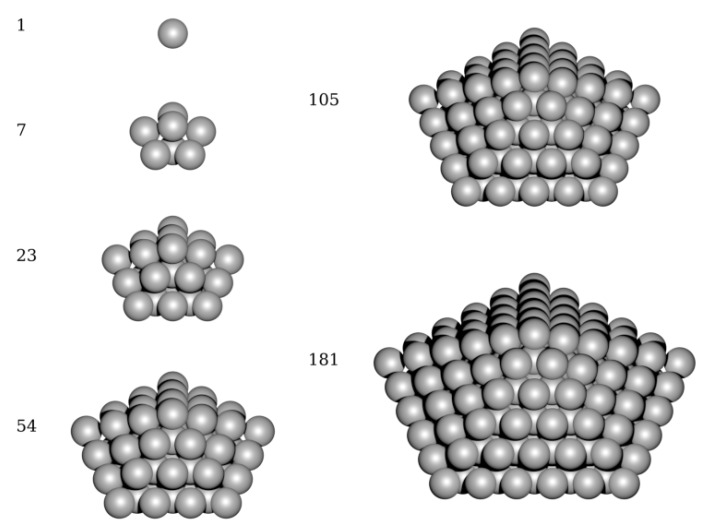
A schematic visualization of studied decahedron nanoclusters and nanoparticles (for higher number of atoms) with each of them accompanied by the number of atoms.

Our second approach is represented by direct *ab initio* calculation of electronic structure of decahedrons, schematically shown in Figure 2. The quantum-mechanical calculations are very computationally demanding in this case but still feasible for systems of a few hundred of atoms such as the studied decahedral nanoparticles/nanoclusters. Each of the computed nanoclusters/nanoparticles was treated inside a larger computational supercell where it was surrounded by vacuum, but the periodic boundary apply to these supercells. The positions of atoms in the nanoclusters were optimized so as to minimize the total energy which is provided by our *ab initio* software package (see Section 2.2). The total energy includes electronic-structure energy terms such as the Hartree energy, exchange-correlation energy, local ionic pseudopotential energy or kinetic energy as well as Madelung energy of the ions. The total energy is essentially related to T = 0 K without any entropy contributions and its minimum corresponds to the ground state of each nanocluster/nanoparticle.

### 2.1. Phenomenological Thermodynamic Modeling

The phenomenological thermodynamic approach based on the CALPHAD method is very often used for calculations of the total energy of particles as well as for the prediction of phase diagrams [22,23,24,25,26,43]. The computations use an approximation when bulk variables are applied in the case of nanoparticles but not all properties of the (nano-)particles are included (for example, a structural disorder is sometimes omitted). The molar total Gibbs energy $G_{\mathrm{tot}}$ is decomposed into a sum of relevant contributions [24]:

(1) $$G_{\mathrm{tot}}=G_{\mathrm{ref}}+G_{\mathrm{id}}+G_{E}+G_{\mathrm{mag}}+G_{P}+G_{\mathrm{sur}},$$

where $G_{\mathrm{ref}}$ is the molar reference Gibbs energy, $G_{\mathrm{id}}$ is the molar energy of ideal mixing of an alloy, $G_{E}$ is the molar excess Gibbs energy, $G_{\mathrm{mag}}$ is the molar contribution related to magnetism (which could be particularly complicated in the case of magnetic nanoparticles, including spin and orbital moment contributions as discussed, e.g., in Ref. [44]), $G_{P}$ accounts for the influence of pressure *P* and $G_{\mathrm{sur}}$ for the molar contribution of surface energy. When adapting the Equation (1) to the studied case of silver nanoclusters/nanoparticles, i.e., an unary non-magnetic metal at the temperature T = 0 K, only the reference Gibbs energy $G_{\mathrm{ref}}$ and the surface Gibbs energy $G_{\mathrm{sur}}$ remain:

(2) $$G_{\mathrm{tot}}=G_{\mathrm{ref}}+G_{\mathrm{sur}}.$$

The surface energy contribution is in the case of spherical nanoparticles equal to:

(3) $$G_{\mathrm{sur}}=\frac{A_{\mathrm{sph}}}{n}\cdot \sigma_{\mathrm{sur}}=\frac{A_{\mathrm{sph}}}{n}\cdot \frac{V_{\mathrm{sph}}}{V_{\mathrm{sph}}}\cdot \sigma_{\mathrm{sur}}=3\cdot \frac{V_{m}}{r}\cdot \sigma_{\mathrm{sur}},$$

where $\sigma_{\mathrm{sur}}$ is surface energy, $A_{\mathrm{sph}}=4\pi r^{2}$ is the surface area of a spherical nanoparticle with the volume $V_{\mathrm{sph}}=\left(4/3\right)\pi r^{3}$, *n* is is the number of moles and the $V_{m}$ is the molar volume. As the volume of nanoclusters/nanoparticle is ill-defined, we below discuss three different ways of assigning the (molar) volume to the studied nanoclusters/nanoparticles. The radius *r* is then set equal to the radius of a sphere, which has the volume equal to the product of the number of particles in the nanocluster/nanoparticle and the (specifically assigned) volume (per atom). The surface area (to be multiplied by $\sigma_{\mathrm{sur}}$) is then put equal to the surface of a sphere with the radius equal to the above discussed radius *r*. The fact, that the shape of studied nanoclusters/nanoparticle is non-spherical, decahedral, is taken into account by a shape factor *C* is introduced [25,43,45] into the Equation (3):

(4) $$G_{\mathrm{sur}}=3\cdot C\cdot \frac{V_{m}}{r}\cdot \sigma_{s},\;C=A_{\mathrm{shape}}/A_{\mathrm{sph}},$$

where the shape factor *C* is defined as the ratio of the surface area of the calculated nanoparticle $A_{\mathrm{shape}}$ to the surface area of a spherical particle with the same volume. For the decahedron we use

(5) $$A_{\mathrm{dec}}=a^{2}\cdot 5\cdot \sqrt{3}\cdot \sin36^{{}^{\circ}}\cdot \sqrt{1-0.75\cdot {\left(\sin36^{{}^{\circ}}\right)}^{2}}\;\mathrm{and}\;V_{\mathrm{dec}}=a^{3}\cdot \left(5/4\right)\cdot \sin36^{{}^{\circ}}\cdot \cos36^{{}^{\circ}}$$

with the length parameter *a* defined in Figure 1 (also equal to the height of the decahedron, a pentagonal dipyramid). The values for a few commonly occurring shapes of (nano-)particles are listed in Table 1. Importantly, following the procedure described above we do not need a Tolman length to define the surface of particle [46] as it is defined by molar volume and radius of a spherical particle.

Analogous to the Equations (2) and (4) is the total Gibbs energy of cluster, $g_{\mathrm{tot}}$ equal to:

(6) $$g_{\mathrm{tot}}=\frac{G_{\mathrm{tot}}}{N_{A}}\cdot N=g_{\mathrm{ref}}+g_{\mathrm{sur}}\;\mathrm{where}\;g_{\mathrm{ref}}=N\cdot g_{\mathrm{Ag}}^{\phi }$$

where *N* is the number of atoms in a studied nanocluster/nanoparticle, $N_{A}$ is the Avogadro constant and the $g_{\mathrm{Ag}}^{\phi }$ is the atomic Gibbs energy of pure constituent Ag. As far as the Gibbs energy $g_{\mathrm{Ag}}^{\phi }$ is concerned, we use the Gibbs energy of the bulk fcc Ag per atom $E_{\mathrm{bulk}}$, calculated by DFT.

The equation of surface contribution for one nanocluster/nanoparticle is then changed to:

(7) $$g_{\mathrm{sur}}=N\cdot 3\cdot C\cdot \frac{V_{\mathrm{at}}}{r}\cdot \sigma_{\mathrm{sur}}=N\cdot 3\cdot C\cdot \frac{V_{m}}{r\cdot N_{A}}\cdot \sigma_{\mathrm{sur}}.$$

where $V_{\mathrm{at}}$ corresponds to the volume of one atom. One of the consequences of a high surface Gibbs energy is a surface strain [17]. It is caused by the minimization of the surface energy of the studied (nano-)particles and it leads to the reduction of the mean molar volume of the nanoparticle. As an extreme case, a particle without any surface energy exhibits zero surface strain and its volume is equal to that of the bulk.

Our phenomenological approach of calculating thermodynamic properties of silver nanoclusters/nanoparticles consisting of *N* atoms at temperature T = 0 K is based on the following procedure. First, the Gibbs energy of the studied nanoparticle has the two contributions mentioned in Equation (6), i.e., $g_{\mathrm{tot}}=g_{\mathrm{ref}}+g_{\mathrm{sur}}$. Importantly, when evaluating the reference energy $g_{\mathrm{ref}}=N\cdot g_{\mathrm{Ag}}^{\phi }$ of the Ag nanoparticles we put the $g_{\mathrm{Ag}}^{\phi }$ equal to the reference Gibbs energy of the bulk $E_{\mathrm{bulk}}$ (per atom), here fcc Ag, which we get from *ab initio* calculations.

All the changes, which are related to the fact, that we assess nanoparticles (and not the bulk), are included in the molar volume of the studied nanoparticles $V_{m}$ and the surface stress $P_{\mathrm{sur}}$, i.e., in the surface energy term $G_{\mathrm{sur}}$:

(8) $$G_{\mathrm{sur}}=P_{\mathrm{sur}}\cdot V_{m}\;\mathrm{where}\;P_{\mathrm{sur}}=\frac{G_{\mathrm{sur}}}{V_{m}}=\frac{3\cdot C\cdot \sigma_{\mathrm{sur}}}{r}.$$

**Figure 3:**
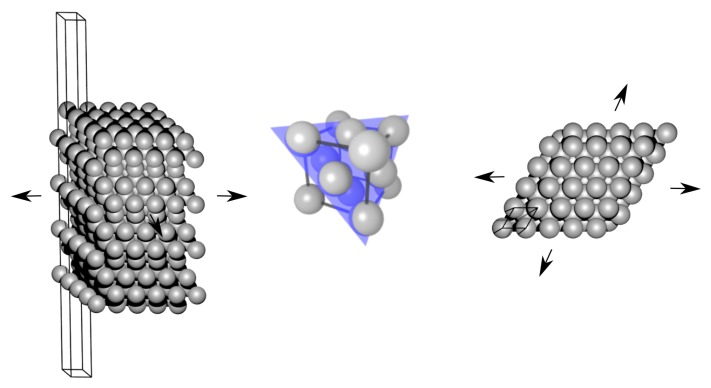
A schematic visualization of a computational cell, so-called slab, used for calculations of surface energy and surface stress in fcc-structure Ag (the visualization shows a 5 × 5 multiple of the studied primitive cell within the surface plane, a side view on the left, and a top view on the right). The surface is formed by the (111) crystallographic plane (see it in the middle also visualized inside a bulk fcc structure elementary cell).

Two further approximations are made. First, the surface stress $P_{\mathrm{sur}}$ is evaluated for each relevant surface orientation, i.e., those existing on the facets of the studied nanoparticles, from the DFT calculations of infinite planar surfaces of the bulk system—see its schematic visualization in Figure 3. It means that we use a bulk-related property, the surface stress, instead of the surface stress (or surface energy) which would be related to any actual nanoparticles (there the surfaces contain edges and vertices where individual facets meet). In our particular case of decahedral nanoparticles, which have only {111} facets, our DFT calculations were performed for the (111) surface of fcc Ag.

The second important approximative step is related to the evaluation of the molar volume of the studied nanocluster/nanoparticle. In order to assign it to a particular decahedral nanoparticle, we put the surface stress $P_{\mathrm{sur}}$ equal to a fictitious hydrostatic pressure *p* which would be acting on every atom of the studied nanoclusters/nanoparticles. We thus do not take into account any elastic strains and stresses which are inside of decahedral nanoparticles due to the unfilled gap of 7.35°. Instead, we apply the surface stress to all particles as if it were a hydrostatic pressure *p* acting upon all atoms in the nanoparticle. For calculations of the molar volume of the particle (from the known molar volume of a bulk system) we apply the following three methods.

First, we use the Murnaghan equation [50,51] applied to the bulk:

(9) $$p\left(V_{m}\right)=\frac{B_{0}}{B_{0}^{\prime }}\cdot \left[{\left(\frac{V_{m,0}}{V_{m}}\right)}^{B_{0}^{\prime }}-1\right]\;\mathrm{and}\;\mathrm{we}\;\mathrm{put}\;p\left(V_{m}\right)=P_{\mathrm{sur}}.$$

The Murnaghan equation of state above contains the following (bulk-related) quatities: $B_{0}$ is the bulk modulus, $B_{0}^{\prime }$ is its pressure derivative and $V_{m},0$ is the molar volume of the bulk material. The values of $B_{0}$, $B_{0}^{\prime }$ and $V_{m},0$ are obtained from energy-volume curve of *ab initio* calculation of the bulk fcc Ag. The equation allows to assign the molar volume $V_{m}$ to the studied nanocluster/nanoparticle.

The second way of assigning a volume to the studied nanoclusters/nanoparticles is based on the definition of the bulk modulus $B_{0}$ [52] where we use finite differences instead of derivatives:

(10) $$B_{0}=-V_{m}\cdot \frac{dp}{dV_{m}}\approx -V_{m}\cdot \frac{\Delta p}{\Delta V_{m}}=-Vm\cdot \frac{0-p(V_{m})}{V_{m},0-V_{m}},\;\mathrm{and}\;\mathrm{where}\;\mathrm{we}\;\mathrm{put}\;p\left(V_{m}\right)=P_{\mathrm{sur}}$$

and there is one state of the bulk for which $p(V_{m})$ is equal to 0, $V_{m}$ is the value of bulk molar volume and the second state is such that there is a non-zero pressure $p(V_{m})$ applied on the particle. Instead of extrapolating from $V_{m},0$ using $B^{\prime }$ from the Murnaghan equation as in Equation (9), we put $p(V_{m})$ equal to $P_{\mathrm{sur}}$ and determine the volume directly from Equation (10). Again, the bulk-related quantities, such as the bulk modulus $B_{0}$ and the molar volume of the bulk material $V_{m},0$ are determined from quantum-mechanical calculations of bulk fcc Ag.

Third, in the following section we also consider the case when the volume of the atoms in the studies nanoclusters/nanoparticles is simply set to be equal to the volume of atoms in the bulk fcc Ag, i.e., the molar volume is not affected by the fact that we study a nanocluster/nanoparticle system.

The above described series of approximative steps, which we apply as a part of our phenomenological thermodynamic approach to nanoparticles, is tested by direct quantum-mechanical calculations of energies of static (T = 0 K) atomic configurations of a series of nanoclusters/nanoparticles visualized in Figure 2. The energy of these nanoclusters/nanoparticles is computed directly and compared to the results of the phenomenological thermodynamic approach described above (including three different ways of assigning the molar volume to the studied nanoparticle).

### 2.2. Parameters of Our DFT Calculations

All our DFT calculations were performed using the Vienna Ab-initio Simulation Package (VASP) [53,54]. The exchange and correlation energy was treated in the generalized gradient approximation (GGA) as parametrized by Perdew, Burke and Ernzerhof (PBE-96) [55]. The used Ag pseudopotential contains 1 *s* electron and 10 *d* electrons. We prefer the GGA-PBE exchange-correlation approximation over the the local density approximation (LDA) [21] because the former gives the value of the bulk modulus of silver closer to the experimental value (see the discussion below). Consequently, we assumed a better description of strained/stressed states. The cut-off plane-wave energy was equal to 550 eV and the employed spacing between k-points amounted to 0.11 Å${}^{-1}$. When minimizing the total energy, the forces acting upon atoms of the surface slabs were reduced under 0.01 meV/Å while those acting upon atoms of the nanoparticles were minimized under 0.1 meV/Å.

The surface energies and stresses were determined from DFT calculations employing computational supercells with so-called slab geometry, see an example for the (111) surface [56] in Figure 3. The surface energy of an infinite slab $\sigma_{\mathrm{hkl}}$ (where {hkl} are mainly {111}, {100} and {110} for fcc-structure faces) was calculated as a difference of the relaxed surface energy $E_{\mathrm{sur}}\left(N\right)$ and the relaxed bulk $E_{\mathrm{bulk}}\left(N\right)$ per surface area *S*:

(11) $$\sigma_{\mathrm{hkl}}=E_{\mathrm{sur}}\left(N\right)-E_{\mathrm{bulk}}\left(N\right)/2\cdot S,$$

with both energies being related to systems with the same number of atoms *N*.

Due to various shape of nanoparticles, the mean surface energy of nanoclusters/nanoparticles is computed according to the approach suggested by Guisbiers and Abudukelimu in [57]

(12) $$\sigma_{\mathrm{sur}}=\left(\sum A_{\mathrm{hkl}}\cdot \sigma_{\mathrm{hkl}}\right)/\sum A_{\mathrm{hkl}},$$

where $A_{\mathrm{hkl}}$ are areas of facets with different {hkl} crystallographic orientation on the surface of a nanocluster/nanoparticle. In the following, we put the energy $\sigma_{\left\{111\right\}}$ equal to the surface energy $\sigma_{\mathrm{sur}}$ of the whole decahedron particle as its surface contains only the {111} facets.

**Figure 4:**
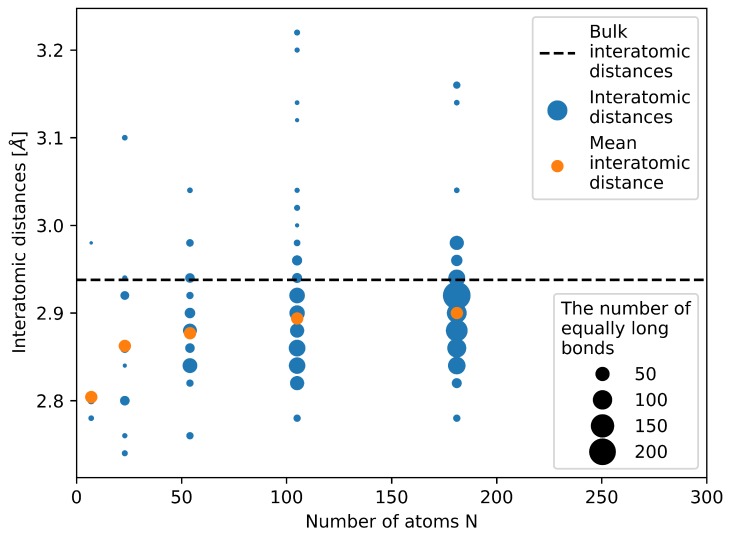
Computed bond lengths between pairs of nearest neighbors in the studied nanoclusters/nanoparticles. Blue symbols represent all inter-atomic distances in calculated nanoparticles, orange color marks the mean inter-atomic distances and the black dashed line shows the inter-atomic distance of bulk fcc structure of Ag.

Next to the energy we also make an attempt to determine the molar volume of the studied nanoparticles from our quantum-mechanical calculations by the following steps. We first compute the mean radius as a half of the average inter-atomic distance between all the atoms and all their nearest neighbors—see Figure 4. Second, we put this mean radius equal to a radius of equally sized touching spheres in a fcc bulk crystal (as when computing the atomic packing factor, for fcc equal to 0.74). Third, we assign the atomic volume in such a fcc bulk crystal to each atom in our nanocluster.

The figure neatly shows that the studied nanoclusters/nanoparticles are highly strained. The majority of bond lengths (interatomic distances) is well below the bulk value of fcc Ag (see the horizontal black dashed line in Figure 4). In particular, this is true for the two nanoclusters with the number of atoms equal to 7 and 23. The mean (average) interatomic distance (see the orange data points) clearly demonstrates this reduction of the interatomic distances. It is worth noting that internal elastic strains (and the corresponding energies) are not included in our approximative phenomenological thermodynamic description of nanoclusters/nanoparticles (as described in the subsection above) but all particles are subject to a fictitious hydrostatic pressure (which we put equal to the surface stress value).

## 3. Results and Discussion

**Figure 5:**
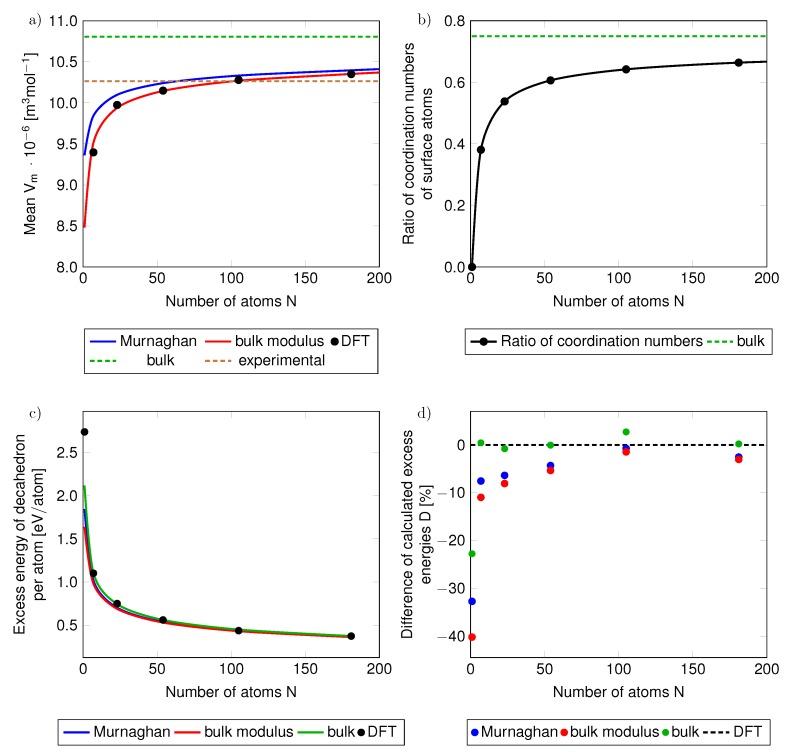
The dependencies of (**a**) the molar volume and (**b**) the ratio of coordination numbers of nanocluster surface atoms with respect to that of {111} surface of the bulk (which is equal to 9) as functions of the number of atoms in the studied nanoclusters/nanoparticles. The green dashed lines in parts (**a**,**b**) are the values corresponding to the bulk (or its {111} surface), blue and red lines represent volumes assigned to nanoparticles using Murnaghan equation and the definition of the bulk modulus, respectively. The full black circles represent results of direct DFT calculations of nanoparticles. The part (**c**) shows absolute (and part (**d**) also relative) differences of excess energies per atom determined by our phenomenological thermodynamic approach w.r.t. to the excess energies of the direct DFT calculations of nanoparticles when the volume of nanoparticles in the phenomenological thermodynamic modeling is determined from the Murnaghan equation (blue curves and blue data points in part (**d**)), from the definition of the bulk modulus (red curves and red data points) and from determining the volume of the nanoparticles from the volume of bulk fcc Ag (green lines and green data points). Also added is the experimental molar volume (the horizontal brown dashed line in (**a**)).

The molar volumes determined from direct quantum-mechanical calculations using the procedure of averaging the interatomic distances (see Figure 4) are compared with those determined from the Murhaghan equation and the definition of the bulk modulus within our thermormodynamic approach in Figure 5a. The volumes obtained from the direct calculations of the electronic structure of nanoclusters/nanoparticles are represented by the DFT black data points, and the molar volumes from our phenomenological thermodynamic approach are continuous blue and red lines for the volumes based on the Murnaghan equation and the definition of the bulk modulus, respectively. It is evident that the volumes from direct DFT calculations of nanoclusters/nanoparticles agree very well with those based on the definition of the bulk modulus (Equation (10)).

In order to determine the molar volumes from the Murnaghan equation of state and the definition of the bulk modulus (shown in Figure 5a) we used the hydrostatic pressure equal to the surface stress $p=P_{\mathrm{sur}}$ which was found from the calculations of the surface energy of Ag for T = 0 K for the {111} and {100} terminations of the bulk fcc Ag. The obtained value of the surface energy for the {111} facet is equal to 0.80 ${\mathrm{Jm}}^{-2}$ and for the {100} surface orientation to 1.14 ${\mathrm{Jm}}^{-2}$. Our values agree quite well with the experimental mean surface energy of 1.1–1.3 ${\mathrm{Jm}}^{-2}$ (see Table 2), reported for much higher temperature of 1073 K in Ref. [58], or with the theoretical values obtained using the LDA approximation in Ref. [59].

Our calculations also reproduce fairly well the lattice constant and the bulk modulus of the bulk fcc Ag. Our theoretical lattice constant of fcc Ag is equal to 4.1555 Å in an acceptable agreement with the experimental value of 4.0853 Å. Our computed bulk modulus of 90 GPa lies between the experimental values of 84 GPa and 118 GPa [60].

In a similar way we analyze also the ratio of the coordination number of surface atoms of the studied nanoparticles, the coordination number of an fcc bulk lattice is 12. The coordination number of the surface atoms is lower. For an infinite surface of a bulk fcc (see the schematics in Figure 3) it is equal to 9 and so the ratio of the coordination numbers of surface atoms of the bulk with respect to the coordination number of atoms in the fcc bulk is 0.75 (see this value as the horizontal green dashed line Figure 5b). The coordination numbers of surface atoms at the {111} facets of the studied nanoclusters/nanoparticles apparently converge to the coordination number of surface atoms at the {111} surface of the bulk only very slowly as a function of the number of atoms in the nanoparticle.

Using our computational approaches it is now possible to evaluate an energy contribution related to the fact that the studied systems are nanoclusters/nanoparticles (with respect to the energy of the bulk). As this energy has a character of an excess energy $E_{\mathrm{ex}}$ (the total energy of nanoclusters/nanoparticles without the cohesion energy of the bulk):

(13) $$E_{\mathrm{ex}}=\frac{E_{\mathrm{tot}}-N\cdot E_{\mathrm{bulk}}}{N}$$

we show it (per atom) in Figure 5c as a function of the number of atoms in the studied nanoclusters/nanoparticles. The excess energy per atom decreases with increasing radius of nanoparticles. Let us note that this excess energy is different from the excess Gibbs energy $G_{E}$ employed in Equation 1, similarly as in other papers dealing with nanoparticles, e.g., Ref. [61].

While Figure 5c clearly shows that the absolute values of the excess energies (per atom) as determined using (i) our phenomenological thermodynamic approach based on bulk-related properties (obtained by DFT calculations) very well match (ii) those from direct DFT calculations of actual nanoclusters/nanoparticles $E_{\mathrm{ex}}^{\mathrm{DFT}}$, it is important to evaluate the differences more precisely. Therefore, we analyze the excess energy differences as relative values:

(14) $$D=\frac{E_{\mathrm{ex}}-E_{\mathrm{ex}}^{\mathrm{DFT}}}{E_{\mathrm{ex}}^{\mathrm{DFT}}}\cdot 100\%.$$

The results are presented in Figure 5d again for differently defined volumes of nanoclusters/nanoparticles. Importantly, the Figure 5d clearly demonstrates that when using the phenomenological thermodynamic approach based on (i) the bulk reference energy, (ii) the bulk surface stress (slab calculations in Figure 3) and (iii) the bulk atomic volume, then the relative differences with respect to the energies obtained from direct DFT calculations of nanoclusters are only a few %, see the green data points in Figure 5d. The only exception from this nice agreement is the limiting case of a single silver atom (the relative error of the excess energy is over −22%). The actual values of the relative differences of the excess energy are summarized in Table 3.

The agreement can be interpreted so that that the surface-related energy of the phenomenological thermodynamic model of static configuration of atoms in a nanocluster/nanoparticle at T = 0 K (blue, red and green data points in Figure 5d) covers a vast majority of the excess energy which is determined by the DFT calculations of the actual nanoclusters (black horizontal dashed line in Figure 5d). We thus demonstrate that, in the case of the total energy of static atomic configurations of nanoparticles, the top-down phenomenological approach can be extended from the bulk down to nanoclusters containing essentially only a few atoms. The only necessary information related to the nanoparticle of interest is then (i) the number of atoms, (ii) the type of surface facets (their crystallographic orientation) and (iii) the shape of the nanocluster/nanoparticle.

**Figure 6:**
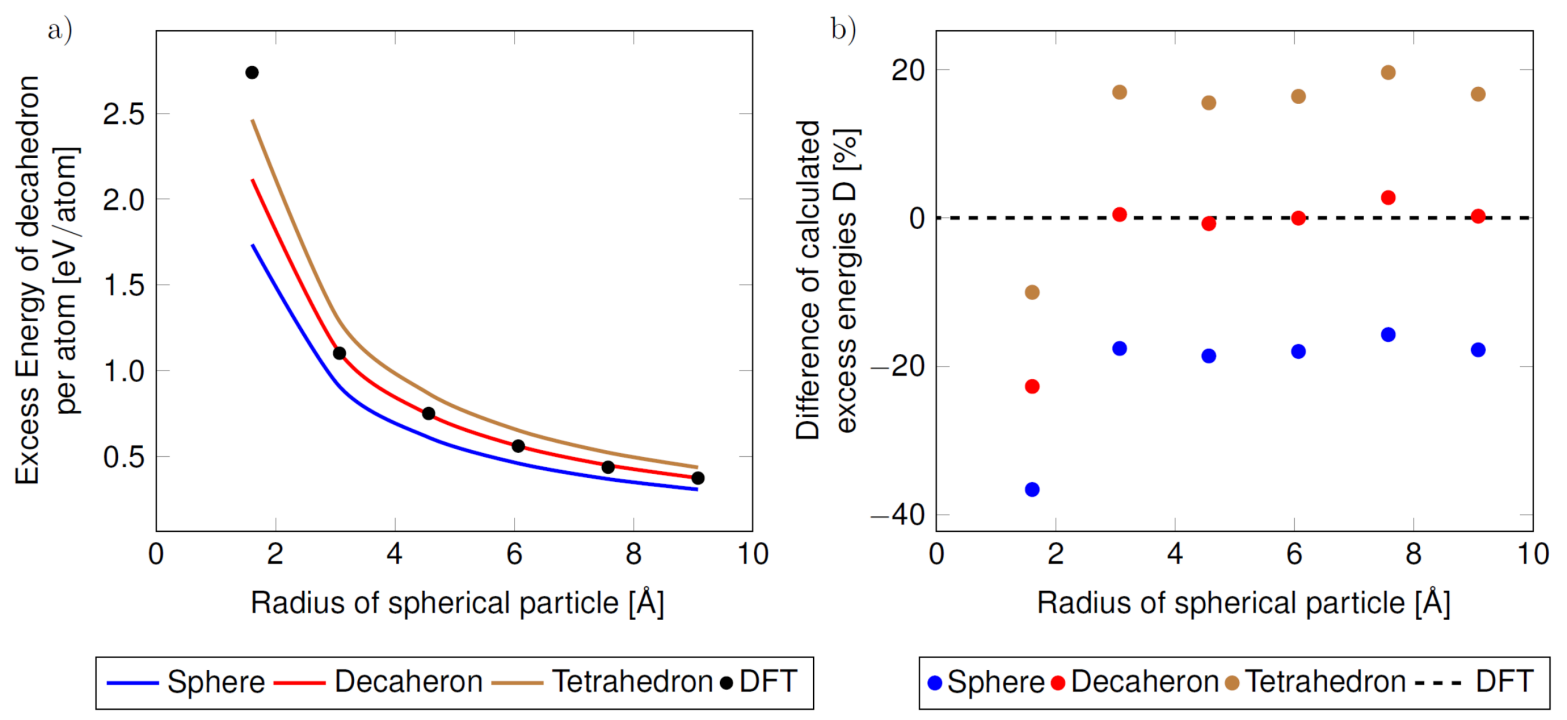
The computed excess energies of the studied nanoclusters/nanoparticles (per atom and as functions of the nanoparticle radius) as obtained from the phenomenological thermodynamic modeling when considering different shapes of the nanoparticles with the same number of atoms. The absolute excess energies are shown in part (**a**) and compared with the excess energies from the direct DFT calculations of the actual decahedral nanoparticles (shown as black symbols in part (**a**)). The differences of the excess energies are presented also relatively in part (**b**) with respect to the DFT values (horizontal black dashed line). The blue curve in part (**a**) and blue data points in part (**b**) correspond to the spherical shape, red to the decahedral shape and brown to the tetrahedral shape.

The last aspect enters our phenomenological approach via the shape factor *C* (see Equation (4) and Table 1). Its importance is demonstrated in Figure 6 where the predictions of the phenomenological thermodynamic modeling are visualized for the same set of DFT values related to the bulk but for different values of the shape factor *C* corresponding to differently shaped nanoparticles. For a spherical nanoparticle, when the surface is not formed by planar {111} facets as in the case of decahedron, the surface energy was put equal to the average of surfaces energies obtained by DFT calculations of {100} and {111} surfaces.

Regarding the tetrahedron, the {111} surface energy was used similarly as in the case of the decahedron. As seen in Figure 6a,b, the best agreement between the total energies of nanoparticles determined from phenomenological thermodynamic modeling and those obtained from direct DFT calculations of nanoparticles is found when the actual (decahedral) shape of nanoparticles is considered. Our calculations also confirm the previous findings that the surface energy and strain energy change rapidly with the change of structure (accompanying the change of temperature [8]). In agreement with results published by Vollath et al. in Ref. [62] our analysis also demonstrates that changes of surface energy are not noticeable for nanocluster/nanoparticles sizes less than 4 Å.

Finally, it is worth mentioning that our conclusion, that the energy of Ag nanoclusters and nanoparticles can be quite reliably assessed using the volume of the bulk fcc Ag, agrees well with the concept of so-called surface area correction [61,63] which is related to the expansion of the electronic cloud around the nanoclusters/nanoparticles. This phenomenon is specifically important for small nanoparticles and the resulting volume is, in the case of nano-sized systems, put equal to that of the bulk material. Our recommended choice of the volume in the case of nanoclusters/nanoparticles (to be set equal to the volume of the same number of atoms in the bulk) is thus neatly justified also by the electronic structure of the discussed nanoparticles.

## 4. Conclusions

With the help of first-principles calculations, we investigated properties of silver decahedral nanoclusters/nanoparticles containing 1–181 atoms in their static atomic configurations corresponding to the minimum of the quantum-mechanically computed total energies. Our T = 0 K thermodynamic analysis compares excess energies (per atom) obtained from a phenomenological approach, which is mostly based on bulk-related properties, with excess energies of direct quantum-mechanical DFT calculations of actual nanoclusters/nanoparticles. We show that the phenomenological thermodynamic modeling, which uses (i) the bulk reference energy, (ii) surface energies obtained for infinite planar (bulk-related) surfaces and (iii) the bulk atomic volume can predict the excess energy per atom of the studied nanoclusters/nanoparticles with the error lower than 3% with the only exception being the limiting case of a single silver atom. This agreement is achieved when the bulk-related properties (the bulk reference energy, the atomic volume and surface energy) are determined by the *ab initio* calculations performed as much as possible on equal footing with direct quantum-mechanical calculations of the studied nanoclusters/nanoparticles, i.e., with the same computational parameters (the same exchange-correlation functional, energy cut-off, k-point density,…). The only necessary information related to the nanoclusters/nanoparticles of interest, which enters the phenomenological thermodynamic modeling, is the number of atoms in the nanocluster/nanoparticle, their shape and the crystallographic orientations of facets. Importantly, the quantum-mechanical calculations of bulk-related properties are much less computationally demanding and we demonstrate that a top-down phenomenological approach can be extended from the bulk down to nanoclusters containing only a few atoms.

Our work thus indicates that it is possible to substantially reduce computational demands when assessing thermodynamic properties of nanoclusters and nanoparticles by quantum-mechanical methods. We would also like to emphasize that, importantly, the agreement between (i) our phenomenological modelling and (ii) the DFT energies for the actual nanoclusters has not been found sensitive to minor deviations of the shape of the studied nanoclusters from a geometrically ideal decahedral case (due to atomic relaxations in our DFT calculations). On the other hand, it should be noted that (i) our study does not cover any excitations, such as phonons, and (ii) whenever the absolute value of the excess energy, i.e., not per atom, is needed when thermodynamically assessing the stability of nanoclusters/nanoparticles, the deviation of the absolute excess energies as obtained from our method may change with the number of atoms (with respect to absolute excess energies from direct *ab initio* calculations of the studied nanoclusters/nanoparticles).

## Figures and Tables

**Table 1:** Shape factors for different shapes of (nano-)particles as collected from selected literature sources. By a liquid spherical shape we mean an ideal sphere (without any atomic structure manifesting itself) while solid spherical shape represents a spherical nanoparticle with its atomic structure which is making its surface not ideally spherical.

Shape of Particle	Shapefactor	References
spherical - liquid	1.00	[12,22,47,48]
spherical - solid	1.05	[22,47,48]
regular icosahedron	1.06	[12,49]
regular dodecahedron	1.10	[49]
regular octahedron	1.18	[12,49]
cube	1.24	[12,49]
decahedron	1.28	this work
regular tetrahedron	1.49	[12,43]

**Table 2:** Our computed surface energies for Ag surfaces with different crystallographic orientations in comparison with available experimental data [58].

	eV/atom	J/m${}^{2}$
(111)	0.409	0.881
(100)	0.646	1.206
(110)	0.801	1.057
exp. [58] (1073 K)		1.1–1.3

**Table 3:** Relative differences (Equation (14)) of the excess energy per atom (Equation (13)) shown in Figure 5 and Figure 6.

D [%] of the Excess Energy $E_{\mathrm{ex}}$ Per Atom	Number of Atoms in the Nanocluster/Nanoparticle					
	1	7	23	54	105	181
from the Murnaghan in Figure 5d	−32.7	−7.5	−6.3	−4.3	−0.7	−2.5
from the bulk modulus in Figure 5d	−40.1	−10.9	−8.0	−5.3	−1.4	−3.0
from the bulk value in Figure 5d	−22.7	0.5	−0.8	0.0	2.7	0.2
spherical shape in Figure 6d	−36.6	−17.6	−18.6	−18.0	−15.7	−17.8
decahedral shape in Figure 6d	−22.7	0.5	−0.8	0.0	2.7	0.2
tetrahedral shape in Figure 6d	−10.0	16.9	15.5	16.4	16.6	16.7
